# Microbiome-mediated colonization resistance to and countermeasures of *Klebsiella pneumoniae*

**DOI:** 10.1038/s41467-026-69690-9

**Published:** 2026-02-16

**Authors:** Yongqiang Yang, Willem van Schaik, Alan McNally, Zhiyong Zong

**Affiliations:** 1 Center of Infectious Diseases, West China Hospital, Sichuan University, Chengdu, China; 2 Department of Microbes, Infection and Microbiomes, School of Infection, Inflammation and Immunology, College of Medicine and Health, University of Birmingham, Birmingham, UK

**Keywords:** Bacterial pathogenesis, Microbiome

## Abstract

Yang *et al*. summarize that *Klebsiella pneumoniae* affects infectious and non-infectious disease via gut colonization; commensals provide colonization resistance, but *K. pneumoniae* adapts via multiple mechanisms.

## Teaser

*Klebsiella pneumoniae* intestinal colonization contributes to infectious and non-infectious diseases. Recent studies have uncovered the complex interplay between the gut microbiota and *K. pneumoniae* colonization, highlighting its role in disease development and progression and illustrating the potential of microbiome-based therapies.

## *Klebsiella pneumoniae* gut colonization contributing to diseases

*Klebsiella pneumoniae* is one of the ESKAPE pathogens, posing a dual threat due to its extensive antimicrobial resistance and virulence, which together impose substantial clinical and economic burdens^1^. *K. pneumoniae* is one of the leading causes of respiratory tract infections (pneumonia), urinary tract infections (cystitis, pyelonephritis), bloodstream infections, central nervous system infections (meningitis), wound and surgical site infections, liver abscesses, intra-abdominal infections, and ocular infections^1^. Capsule, lipopolysaccharide, and fimbriae are key virulence and colonization determinants that confer *K. pneumoniae* with advantages against bacterial competition, complement-mediated clearance, and host immune evasion^2^. Notably, beyond its established role in severe infections, *K. pneumoniae* is increasingly recognized as a contributor to non-infectious diseases, and its presence has been correlated with inflammatory bowel diseases^3^, non-alcoholic fatty liver disease^4^, hepatocellular carcinoma^5^, and auto-brewery syndrome^6^. The gastrointestinal tract constitutes the primary reservoir of *K. pneumoniae*, and intestinal colonization represents a pivotal risk factor to both local and systemic diseases. Intestinal colonization or outgrowth has been reported to directly contribute to the development of subsequent infections^7^ and various non-infectious diseases^3,5^.

## Microbiome-mediated colonization resistance against *K. pneumoniae*

The gut microbiota in healthy individuals harbors an ecosystem with a high microbial diversity, including numerous commensal bacteria that collectively confer colonization resistance against pathogenic invasion^8^. Identifying key commensals capable of inhibiting *K. pneumoniae* gut colonization has important implications for both therapeutic and preventive strategies.

Two main approaches are applied to identify such commensals, including direct screening from the microbiota of healthy donors and reverse identification of species with reduced abundance in hosts with gut dysbiosis. Colonization resistance can be evaluated either by in vitro inhibition of *K. pneumoniae* growth or by in vivo murine decolonization models. A coculture competition assay involving 100 human gut bacterial isolates against *K. pneumoniae* has revealed that single species provided no robust colonization resistance, while resistance required a diverse community, dependent on specific compositions and interactions^9^. A defined consortium of 10 species was able to suppress *K. pneumoniae* growth by >1000-fold in vitro^9^. In another study, human fecal microbiota transplantation (FMT) samples that could effectively decolonize *K. pneumoniae* in a mouse model with a humanized microbiota were used to isolate commensal bacteria, with further optimization yielding an 18-member consortium (F18-mix). In germ-free mice monocolonized with carbapenemase-producing *K. pneumoniae*, oral inoculation of F18-mix reduced its levels in feces by three orders of magnitude by day 7 and maintained the effect through day 28^10^.

In contrast, in dysbiotic hosts such as patients in intensive care units whose gut microbiota are severely disrupted due to intensive medical interventions, colonization resistance is compromised, allowing the outgrowth of carbapenem-resistant *K. pneumoniae* (CRKP). Both in vitro and in vivo studies have demonstrated that probiotic supplementation of *Lactiplantibacillus plantarum* 21790 and *Bifidobacterium longum* 6188 contributed significantly to CRKP decolonization^11^. Notably, decolonized subjects exhibited ecological recovery marked by increased microbial diversity and enrichment of key commensals, including *Bacteroides dorei, Bifidobacterium bifidum, Bifidobacterium pseudocatenulatum*, and *Faecalibacterium prausnitzii*, all of which are also known to enhance colonization resistance and thus support CRKP clearance^12^. However, determining the precise combinations of commensals against different *K. pneumoniae* lineages remains a critical step toward developing probiotic-based therapeutic strategies.

## Microbial interactions conferring colonization resistance

**Fig. 1:**
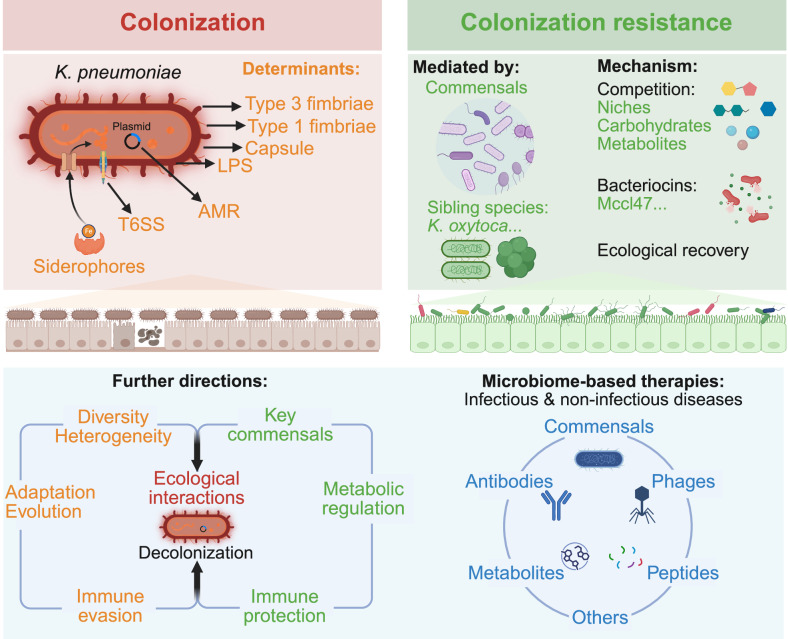
Microbiome-mediated colonization resistance and *Klebsiella pneumoniae* countermeasures. Recent studies have highlighted the important role of *K. pneumoniae* in both infectious and non-infectious diseases, as well as the complex interplay between the gut microbiota and *K. pneumoniae* colonization. In healthy individuals, the gut microbiota establishes colonization resistance against *K. pneumoniae* through balanced ecological niches shaped by key commensals, thereby limiting its colonization. The underlying mechanisms include competitive exclusion for nutrients and ecological niches, secretion of antagonistic molecules, and metabolic interference. However, *K. pneumoniae* can overcome these defenses through intrinsic traits and adaptations, developing multiple strategies to facilitate colonization, such as acquisition of AMR and virulence alterations, unique structural features (e.g., capsule, LPS, and fimbriae), elimination of competitors via T6SS, and metabolic adaptations conferring survival advantages. Future studies should consider the diversity and heterogeneity of *K. pneumoniae* populations, as well as their adaptability and evolutionary dynamics. Research on colonization resistance may focus on the identification of key commensals and their mechanisms of action, including regulation at the metabolic level. In addition, further investigation is needed into the complex interactions between *K. pneumoniae* and other gut microbes, as well as host immune evasion strategies. Microbiome-based therapeutic approaches against *K. pneumoniae*, including commensals, phages, peptides, metabolites, and antibodies, represent promising candidates. AMR antimicrobial resistance, LPS lipopolysaccharide, T6SS the type VI secretion system. Created in BioRender. Yang, Y. (2026) https://BioRender.com/ytu7xgk.

Key commensals employ diverse competitive strategies to suppress *K. pneumoniae* colonization, including nutrient competition, production of inhibitory metabolites, and stimulation of host immune defenses (Fig. 1). In murine models, *Klebsiella oxytoca* suppresses *K. pneumoniae* colonization by facilitating the functional recovery of beneficial commensal bacteria, thereby restoring colonization resistance in a diet-dependent manner^13^. Mechanistically, *K. oxytoca* exhibits a broader carbon utilization spectrum that overlaps with *K. pneumoniae*, thereby limiting its invasion^14^. Specifically, protective *K. oxytoca* strains were more adapt at utilizing β-glucosides and thus outcompeted *K. pneumoniae* in vitro and in mice^14^. Three additional bacterial strains, *Blautia coccoides* YL58*, Enterococcus faecalis* KB1*, and Enterocloster clostridioformis* YL32, were identified to cooperate with *K. oxytoca* for long-term clearance of *K. pneumoniae* strain MDR1 by further restricting access to carbon sources^14^. The importance of carbon sources in the intestinal environment for colonization by *K. pneumoniae* is further supported by the observation that lactulose can enhanced gut colonization, as it is not absorbed by the host and can be metabolized by *K. pneumoniae*^15^. *K. oxytoca* has also been evaluated as a live biotherapeutic, demonstrating the ability to decolonize *K. pneumoniae*, restore gut barrier integrity, and prevent its translocation from the intestine to the liver^5^. Because certain *K. oxytoca* strains can be pathogenic^16^, careful safety evaluation and selection of commensal strains are necessary. *Lactobacillus*-driven expansion of *Clostridiales* also reduces multidrug-resistant *K. pneumoniae* colonization by secreting butyrate and depleting key nutrients such as serine, threonine, and glucose^17^. At the community level, commensal consortia conferring colonization resistance have been found to suppress *K. pneumoniae* by competitively depleting gluconate, a preferential carbon source for *Enterobacteriaceae*, including *K. pneumoniae*, while leaving non-competing commensals unaffected^10^.

Commensals also secrete bactericidal or bacteriostatic compounds against *K. pneumoniae*. For example, class IIb microcin I47 (MccI47), a siderophore-linked antimicrobial peptide produced by *E. coli*, significantly inhibited CRKP in murine models when delivered either as a purified peptide or via an engineered *E. coli* strain Nissle 1917 producing MccI47, without disrupting the resident microbiota^18^. This highlights the potential of narrow-spectrum targeted intervention strategies for selective *K. pneumoniae* suppression. In addition, tilimycin, a DNA-alkylating enterotoxin produced by *K. oxytoca*, functions as a mutagen that increases mutation rates and induces DNA damage of *K. pneumoniae*, thereby facilitating decolonization^19^. Collectively, these findings suggest that targeting *K. pneumoniae*-specific metabolic pathways and leveraging commensal-derived metabolites or nutrient competition may provide novel microbiome-based interventions to prevent or eliminate *K. pneumoniae* colonization.

## *K. pneumoniae* countering colonization resistance

Despite the protective role of the gut microbiota, *K. pneumoniae* has evolved multiple mechanisms to overcome colonization resistance, enabling its expansion in dysbiotic hosts^5^ (Fig. 1). The bacterial type VI secretion system (T6SS) is a phage tail-like contractile nanomachine that mediates contact-dependent killing of bacterial competitors as well as adhesion and invasion of host cells. The T6SS of *K. pneumoniae* is required for robust colonization of the murine gastrointestinal tract under conditions of an intact microbiota. Notably, gut-mimetic conditions, characterized by high arginine availability, low iron, and hypoxia, strongly induce T6SS expression. These signals upregulate transcription and enhance T6SS activity, resulting in the targeted killing of competing Betaproteobacteria and thus facilitating *K. pneumoniae* persistence^20^.

*K. pneumoniae* further facilitates gut colonization by compromising host mucosal defenses. A recent study has shown that *K. pneumoniae* was highly enriched in stool samples from patients with hepatocellular carcinoma, and a similar enrichment was observed in the feces of mice receiving FMT from these patients. Further gavage of isolated *K. pneumoniae* strains to germ-free mice revealed that these strains disrupted colonic intercellular junctions, thinned the protective mucus layer, downregulated tight junction proteins, and enhanced bacterial penetration into the lamina propria and muscularis mucosa^5^. This study provides a new perspective on how *K. pneumoniae* gains a colonization advantage in the gut, translocates via the gut-liver axis, and ultimately contributes to the progression of non-infectious liver diseases. *K. pneumoniae* has some structural features that contribute to its colonization capacity. Type 1 and type 3 fimbriae are commonly associated with adherence to mucosal surfaces^21^; however, the regulatory mechanisms governing bacterial colonization are largely unknown. Notably, regulatory genes can be conserved across *K. pneumoniae* strains, which means modulation of gene expression through a signaling pathway involving environmental stimuli, rather than gene presence or absence, may be a key determinant of colonization fitness. In one study, clinical *K. pneumoniae* strains with high colonization abilities were identified from a murine colonization model and subsequently subjected to in vitro experimental evolution. This approach led to the identification of functional mutations in *cpxA*, encoding the sensor kinase of the CpxR-CpxA two-component system. Mechanistically, CpxA negatively regulates type 3 fimbriae expression, thereby influencing colonization efficiency^22^.

Together, these strategies, including competitive killing via T6SS, specialized surface structures, and direct host barrier disruption, highlight the evolutionary plasticity of *K. pneumoniae* in overcoming colonization resistance and establishing persistent niches in the gut, which can impact the health of the host. These findings also highlight *K. pneumoniae* as a highly heterogeneous population in which distinct strains exhibit divergent regulatory strategies and colonization phenotypes. Such diversity may confer population-level adaptability, enabling different lineages to colonize and persist across host-associated environments.

## Conclusions and future perspectives

The role of *K. pneumoniae* in both infectious and non-infectious diseases receives increasing attention. In infectious contexts, research has focused primarily on antimicrobial resistance and virulence, while in non-infectious diseases focused on metabolic interactions and host immune modulation. Recent findings underscore the complex interplay between colonization-resistant commensals and *K. pneumoniae*. Key commensal species or microbial consortia can suppress *K. pneumoniae* through nutrient and metabolic competition, secretion of effector molecules, and reinforcement of host epithelial and immune barriers. Conversely, *K. pneumoniae* has evolved counterstrategies, including unique cell wall and capsule structures, specialized metabolic pathways, and deployment of the T6SS to evade colonization resistance.

Microbiome-based decolonization is a promising therapeutic approach, but current studies remain limited, and several research gaps need to be considered. First, the genetic and phenotypic diversity of *K. pneumoniae* is rarely addressed, particularly in non-infectious diseases, as only a few studies have examined which specific *K. pneumoniae* lineages are effectively suppressed by colonization-resistant microbiota^3^. Notably, some *K. pneumoniae* types, like hypervirulent sequence type 23 strains with thick capsules, can colonize in the presence of an intact microbiota^23^ and have been detected in the gastrointestinal tracts of healthy individuals^24^. Importantly, the diversity of *K. pneumoniae* must be considered across geographic contexts and host populations, with distinctions between colonizing and infecting strains, as well as between healthy carriers of strains or hospitalized individuals. While healthcare-associated infections are often dominated by a limited number of high-risk clones, the dominant epidemic lineages vary substantially between regions. Moreover, the dynamic replacement and expansion of specific clones represent an underestimated but clinically significant phenomenon. Examples include the decline of ST258 and ST307 in the United States^25^, capsular-type replacement within ST11 strains in China^26^, and the dynamic predominance of multiple sequence types in South Africa^27^. The replacement of dominant clones and emergence of novel clones underscore the need for sustained genomic surveillance.

Second, gastrointestinal carriage of *K. pneumoniae* is a major risk factor for subsequent infection. Systematic monitoring of intestinal colonization in both healthy individuals and patients remains limited. Colonization rates of *K. pneumoniae* in healthy populations were reported as 5–35% in Western countries and 18.8–87.7% in Asian countries^24^. Among hospitalized patients, carriage rates range from 23 to 70%^21^. The lineages of colonizing strains often overlap with those causing clinical infections, indicating that gut-adapted *K. pneumoniae* can retain pathogenic potential. However, clinical *K. pneumoniae* strains undergoing within-host evolution more commonly acquire mutations in virulence genes, including the capsule, lipopolysaccharide, and iron utilization pathways, leading to attenuated virulence while enhancing fitness in extraintestinal niches through evolutionary trade-offs^28^. Furthermore, colonizing bacterial populations can be more genetically diverse than predominantly invasive strains. This raises the question of why some colonizing types do not cause invasive disease. One possible explanation is the selective pressure of antimicrobial agents, which preferentially drives the expansion and dissemination of some high-risk clones. This discrepancy may also reflect complex ecological interactions involving *K. pneumoniae*, including intra-species interactions, interactions with co-colonizing microbes (bacteria, virus, and fungi), and interactions with the host. Specifically, the gut virome, which is dominated by bacteriophages, is established early in life and exhibits greater diversity than the bacteriome. It plays a critical role in shaping the composition of the gut microbiota, affecting pathogen colonization, and also serve as a biomarker distinguishing different human populations^29^. Bacteriophages have been used for intestinal decolonization of *K. pneumoniae*^30^, and phage cocktails or phage-antibiotic synergy may work synergistically to delay phage resistance and provide broader-spectrum efficacy. Taken together, elucidating these mechanisms will be essential for the rational design of microbiome-based decolonization strategies.

Third, the *K. pneumoniae* lineages and the pathogenic mechanisms for infections and non-infectious conditions may be different. Future studies should consider these differences, distinguishing between infection-associated *K. pneumoniae*, which typically involves rapid expansion of defined clones that breach host defenses, and non-infectious contexts, where more diverse lineages coexist with the host and contribute to disease progression via metabolic pathways. Infection-focused studies will require high-resolution, fine-scale analyses beyond sequence type classification, whereas investigations into non-infectious diseases need to study specific metabolic pathways and their host interactions. Third, the identification of key commensals that confer colonization resistance against *K. pneumoniae* requires careful consideration of geographic, population-specific, and disease-specific heterogeneity. Furthermore, these commensals, which could serve as potential biotherapeutics, need to be validated in vivo to guide the development of precise microbiome-targeted therapeutic strategies.

## References

[1] Naghavi, M. et al. Global burden of bacterial antimicrobial resistance 1990-2021: a systematic analysis with forecasts to 2050. *Lancet* **404**, 1199–1226 (2024). 39299261 10.1016/S0140-6736(24)01867-1 PMC11718157

[2] Nguyen, T. N. T., Howells, G. & Short, F. L. How *Klebsiella pneumoniae* controls its virulence. *PLoS Pathog.* **21**, e1013499 (2025). 40953017 10.1371/journal.ppat.1013499 PMC12435677

[3] Federici, S. et al. Targeted suppression of human IBD-associated gut microbiota commensals by phage consortia for treatment of intestinal inflammation. *Cell* **185**, 2879–2898.e2824 (2022). 35931020 10.1016/j.cell.2022.07.003

[4] Yuan, J. et al. Fatty liver disease caused by high-alcohol-producing *Klebsiella pneumoniae*. *Cell Metab.* **30**, 675–688.e677 (2019). 31543403 10.1016/j.cmet.2019.08.018

[5] Wang, X. et al. Gut-liver translocation of pathogen *Klebsiella pneumoniae* promotes hepatocellular carcinoma in mice. *Nat. Microbiol.* **10**, 169–184 (2025). 39747695 10.1038/s41564-024-01890-9 PMC11726454

[6] Xue, G. et al. Three *Klebsiella* species as potential pathobionts generating endogenous ethanol in a clinical cohort of patients with auto-brewery syndrome: a case control study. *EBioMedicine* **91**, 104560 (2023). 37060744 10.1016/j.ebiom.2023.104560 PMC10139882

[7] Tamburini, F. B. et al. Precision identification of diverse bloodstream pathogens in the gut microbiome. *Nat. Med.* **24**, 1809–1814 (2018). 30323331 10.1038/s41591-018-0202-8 PMC6289251

[8] Woelfel, S., Silva, M. S. & Stecher, B. Intestinal colonization resistance in the context of environmental, host, and microbial determinants. *Cell Host Microbe* **32**, 820–836 (2024). 38870899 10.1016/j.chom.2024.05.002

[9] Spragge, F. et al. Microbiome diversity protects against pathogens by nutrient blocking. *Science* **382**, eadj3502 (2023). 38096285 10.1126/science.adj3502 PMC7616675

[10] Furuichi, M. et al. Commensal consortia decolonize Enterobacteriaceae via ecological control. *Nature* **633**, 878–886 (2024). 39294375 10.1038/s41586-024-07960-6 PMC11424487

[11] Yang, J. et al. Microbiome-mediated colonization resistance to carbapenem-resistant *Klebsiella pneumoniae* in ICU patients. *NPJ Biofilms Microbiomes* **11**, 157 (2025). 40783567 10.1038/s41522-025-00791-x PMC12335454

[12] Kang, J. T. L. et al. Long-term ecological and evolutionary dynamics in the gut microbiomes of carbapenemase-producing Enterobacteriaceae colonized subjects. *Nat. Microbiol.* **7**, 1516–1524 (2022). 36109646 10.1038/s41564-022-01221-w PMC9519440

[13] Almasi, E. D. H. et al. *Klebsiella oxytoca* facilitates microbiome recovery via antibiotic degradation and restores colonization resistance in a diet-dependent manner. *Nat. Commun.* **16**, 551 (2025). 39789003 10.1038/s41467-024-55800-y PMC11717976

[14] Osbelt, L. et al. *Klebsiella oxytoca* causes colonization resistance against multidrug-resistant *K. pneumoniae* in the gut via cooperative carbohydrate competition. *Cell Host Microbe* **29**, 1663–1679 e1667 (2021). 34610293 10.1016/j.chom.2021.09.003

[15] Hecht, A. L. et al. Dietary carbohydrates regulate intestinal colonization and dissemination of *Klebsiella pneumoniae*. *J. Clin. Investig.* **134**, e174726 (2024). 38512401 10.1172/JCI174726 PMC11060737

[16] Yang, J. et al. *Klebsiella oxytoca* complex: update on taxonomy, antimicrobial resistance, and virulence. *Clin. Microbiol. Rev.* **35**, e0000621 (2022). 34851134 10.1128/CMR.00006-21 PMC8635272

[17] Djukovic, A. et al. *Lactobacillus* supports Clostridiales to restrict gut colonization by multidrug-resistant Enterobacteriaceae. *Nat. Commun.* **13**, 5617 (2022). 36153315 10.1038/s41467-022-33313-w PMC9509339

[18] Mortzfeld, B. M. et al. Microcin MccI47 selectively inhibits enteric bacteria and reduces carbapenem-resistant *Klebsiella pneumoniae* colonization in vivo when administered via an engineered live biotherapeutic. *Gut Microbes* **14**, 2127633 (2022). 36175830 10.1080/19490976.2022.2127633 PMC9542533

[19] Kienesberger, S. et al. Enterotoxin tilimycin from gut-resident *Klebsiella* promotes mutational evolution and antibiotic resistance in mice. *Nat. Microbiol* **7**, 1834–1848 (2022). 36289400 10.1038/s41564-022-01260-3 PMC9613472

[20] Bray, A. S. et al. *Klebsiella pneumoniae* employs a type VI secretion system to overcome microbiota-mediated colonization resistance. *Nat. Commun.* **16**, 940 (2025). 39843522 10.1038/s41467-025-56309-8 PMC11754592

[21] Bray, A. S. & Zafar, M. A. Deciphering the gastrointestinal carriage of *Klebsiella pneumoniae*. *Infect. Immun.* **92**, e0048223 (2024). 38597634 10.1128/iai.00482-23 PMC11384780

[22] Li, D. et al. Cpx-mediated amino acid sensing diversifies gastrointestinal colonization of *Klebsiella pneumoniae*. *mLife* **4**, 181–192 (2025). 40313974 10.1002/mlf2.70005 PMC12042121

[23] Teo, T. H. et al. Differential mucosal tropism and dissemination of classical and hypervirulent *Klebsiella pneumoniae* infection. *iScience* **27**, 108875 (2024). 38313058 10.1016/j.isci.2024.108875 PMC10835444

[24] Russo, T. A. & Marr, C. M. Hypervirulent *Klebsiella pneumoniae*. *Clin. Microbiol Rev.* **32**, e00001–19 (2019). 31092506 10.1128/CMR.00001-19 PMC6589860

[25] Ikhimiukor, O. O. et al. Clonal background and routes of plasmid transmission underlie antimicrobial resistance features of bloodstream *Klebsiella pneumoniae*. *Nat. Commun.* **15**, 6969 (2024). 39138200 10.1038/s41467-024-51374-x PMC11322185

[26] Wang, R. et al. Increase in antioxidant capacity associated with the successful subclone of hypervirulent carbapenem-resistant *Klebsiella pneumoniae* ST11-KL64. *Nat. Commun.* **15**, 67 (2024). 38167298 10.1038/s41467-023-44351-3 PMC10761919

[27] Heinz, E. et al. Longitudinal analysis within one hospital in sub-Saharan Africa over 20 years reveals repeated replacements of dominant clones of *Klebsiella pneumoniae* and stresses the importance to include temporal patterns for vaccine design considerations. *Genome Med.* **16**, 67 (2024). 38711148 10.1186/s13073-024-01342-3 PMC11073982

[28] Zaborskyte, G., Hjort, K., Lytsy, B. & Sandegren, L. Parallel within-host evolution alters virulence factors in an opportunistic *Klebsiella pneumoniae* during a hospital outbreak. *Nat. Commun.* **16**, 8727 (2025). 41027917 10.1038/s41467-025-64521-9 PMC12485182

[29] Tisza, M. J. et al. Longitudinal phage-bacteria dynamics in the early life gut microbiome. *Nat. Microbiol.* **10**, 420–430 (2025). 39856391 10.1038/s41564-024-01906-4 PMC11790489

[30] Fang, Q. et al. Safety and efficacy of phage application in bacterial decolonisation: a systematic review. *Lancet Microbe* **5**, e489–e499 (2024). 38452780 10.1016/S2666-5247(24)00002-8

